# Interleukin-10 inhibits interleukin-1β production and inflammasome activation of microglia in epileptic seizures

**DOI:** 10.1186/s12974-019-1452-1

**Published:** 2019-03-28

**Authors:** Yi Sun, Jiangjun Ma, Dongfang Li, Pinggan Li, Xiaolin Zhou, Yu Li, Zhanwen He, Lijun Qin, Liyang Liang, Xiangyang Luo

**Affiliations:** 1 Guangdong Provincial Key Laboratory of Malignant Tumor Epigenetics and Gene Regulation, Sun Yat-Sen Memorial Hospital, Sun Yat-Sen University, Guangzhou, 510120 China; 2 Department of Pediatric, Sun Yat-Sen Memorial Hospital, Sun Yat-Sen University, Guangzhou, 510120 China; 3 Department of Molecular Diagnostics, Sun Yat-Sen University Cancer Center, State Key Laboratory of Oncology in South China, Collaborative Innovation Center for Cancer Medicine, Guangzhou, 510060 China

**Keywords:** Epilepsy, Microglia, IL-10, IL-1β

## Abstract

**Background:**

Microglia are important for secreting chemical mediators of inflammatory responses in the central nervous system. Interleukin (IL)-10 and IL-1β secreted by glial cells support neuronal functions, but the related mechanisms remain vague. Our goal was to demonstrate the efficacy of IL-10 in suppressing IL-1β and in inflammasome activation in mice with epileptic seizure based on an epileptic-seizure mouse model.

**Methods:**

In this study, mice in which epileptic seizures were induced by administering picrotoxin (PTX) were used as a case group, and mice injected with saline were employed as the control group. The expression of nucleic acids, cytokines, or signaling pathways was detected by reverse transcription-polymerase chain reaction (RT-PCR), enzyme-linked immunosorbent assay (ELISA), flow cytometry, and Western blotting.

**Results:**

Our results demonstrated that IL-10 inhibits IL-1β production through two distinct mechanisms: (1) Treatment with lipopolysaccharides (LPS) results in IL-10 overexpression in microglia and reduced NLRP3 inflammasome activity, thus inhibiting caspase-1-related IL-1β maturation; (2) next, autocrine IL-10 was found to subsequently promote signal transducer and activator of transcription-3 (STAT-3), reducing amounts of pro-IL-1β.

**Conclusions:**

Our results indicate that IL-10 is potentially effective in the treatment of inflammation encephalopathy, and suggest the potential usefulness of IL-10 for treating autoimmune or inflammatory ailments.

**Electronic supplementary material:**

The online version of this article (10.1186/s12974-019-1452-1) contains supplementary material, which is available to authorized users.

## Background

Epilepsy is a chronic neurological disease characterized by seizures, which is accompanied by fever in 3.2% to 5.5% of children under the age of 5, as a systemic response to infection, inflammation, or stress [1]. The etiology of most generalized epilepsies cannot be determined with certainty, and seizure severity could be genetically determined by disturbances of receptors in the central nervous system. Besides, aberrant neurotransmitter distribution or other metabolic problems could be involved in epileptogenesis. Previous experimental and clinical evidence highlighted the activation of inflammatory pathways in epilepsy [2–7], raising the possibility that this phenomenon may be a common factor contributing to the etiopathogenesis of seizures in various forms of epilepsy with different etiologies.

Recent research in animal models of seizure and epilepsy has demonstrated that brain inflammation is not only a mere hallmark of tissue pathology but plays an active role in sustaining or precipitating seizures, and contributes to cell loss [8]. In experimental models, intracerebral application of interleukin (IL)-1β has been shown to enhance seizure activity [9]. IL-1β is also capable of affecting neuronal viability and injury [10, 11]. An increase in IL-1β, IL-6, and tumor necrosis factor alpha (TNF-α) in microglia and astrocytes is followed by a cascade of downstream inflammatory events which may recruit cells of the adaptive immune system [2, 12]. Many studies have explored the molecular mechanisms behind the process of inflammation increasing tissue excitability, which is a new and promising avenue of research for seizure treatment or prevention. Importantly, inflammation also significantly affects epileptogenesis [13, 14]. Microglia are a population of immune cells of the central nervous system (CNS) that are involved in phagocytosis, antigen presentation, cell proliferation, cell migration, and cytokine production [15]. Previous studies have shown that cultured microglia not only express proinflammatory cytokines such as TNF-α and IL-1β [16] but also produce anti-inflammatory cytokines, e.g., IL-4 and IL-10 [17]. The activation of microglia was described in cerebral tissues from epileptic rodents and humans [18–21].

An animal study indicated that activated microglia release proinflammatory cytokines to decrease the seizure threshold [22]. Therefore, microglia may help generate seizures by releasing, and responding to, endogenous inflammatory mediators, such as IL-1β [23]. However, it is not clear how epilepsy augments inflammasome activation, and what the roles of IL-10 and IL-1β are in the process.

Lipopolysaccharide (LPS), a cell wall component of gram-negative bacteria and a powerful toll-like receptor 4 (TLR4) ligand, resulted in immediate and long-term decreases in seizure threshold [24]. LPS may also induce immediate focal epileptic-type discharges in the mouse neocortex mediated by IL-1β release [25]. Under physiological conditions, the main function of neural cells is to provide protective patrolling of the brain; however, in the presence of LPS, they adopt the characteristics of brain microglia in which caspase-1 activity becomes increased [26, 27]. Caspase-1 is a cysteine protease that converts pro-IL-1β to the active IL-1β, which is then released extracellularly to propagate inflammatory signals. Active caspase-1 will be released and will cleave pro-IL-1β, leading to the ultimate release of the mature form of the proinflammatory cytokine IL-1β [26, 28].

Our results demonstrated that both endogenous and exogenous IL-10 potently downregulated IL-1β in the microglia from mice exposed to epileptogenic injury caused by STAT-3-dependent inhibition of NOD-like receptor family pyrin domain containing 3 (NLRP3) inflammasome activity. Further, we found that IL-10 reduced the amounts of pro-IL-1β. These findings suggested that IL-10 might alleviate symptoms, influencing disease progression in epilepsy patients.

## Methods

### Aims

To investigate the immunoregulatory effect of IL-10 on IL-1β production in mice with picrotoxin (PTX)-induced seizures, we detected the expression of cytokines and transcription factors in microglia of murine brain tissues by reverse transcription-polymerase chain reaction (RT-PCR), enzyme-linked immunosorbent assay (ELISA), fluorescence-activated cell sorter (FACS), and Western blotting. In addition, the correlation of these factors in cells cultured under different conditions was analyzed.

### Reagents and chemicals

PTX, LPS (from *Escherichia coli* O111:B4, catalog number L 4130), and all of the chemicals used in this study were purchased from Sigma-Aldrich (St. Louis, MO, USA). Hank’s balanced salts solution (HBSS), 4-(2-hydroxyethyl)-1-piperazineethanesulfonic acid (HEPES), and Phenol Red were provided by Euroclone (Via Figino, Italy). Dulbecco’s modified Eagle’s medium (DMEM) and fetal bovine serum (FBS) were purchased from Gibco-BRL Technologies (Carlsbad, CA, USA). Fc receptors were blocked with anti-mouse CD16/CD32 purchased from eBioscience (Waltham, MA, USA). Mouse monoclonal anti-CD11b antibody conjugated to phycoerythrin (PE), mouse monoclonal anti-CD45 antibody conjugated to fluorescein isothiocyanate (FITC), and mouse monoclonal anti-Ly-6C antibody conjugated to allophycocyanin (APC) were purchased from Abcam (Cambridge, MA, USA). Mouse monoclonal anti-CD11b antibody conjugated to FITC was purchased from ProSpec–Tany Technogene Ltd. (East Brunswick, NJ, USA; cat. no. ANT-136). Mouse polyclonal antibodies (pAbs), mouse monoclonal antibody (mAb), glyceraldehyde-3-phosphate dehydrogenase (GAPDH), and secondary horseradish peroxidase-conjugated goat anti-mouse antibodies were obtained from Santa Cruz Biotechnology (Dallas, TX, USA). Mouse polyclonal antibodies (pAbs) STAT-1, phospho-STAT-1, STAT-3, phospho-STAT-3, proIL-1β, procaspase-1, and caspase-1 were purchased from Cell Signaling Technology (Danvers, MA, USA). Mouse Ready-SET-Go ELISA kits for murine IL-1β and IL-10 were obtained from BD Bioscience Pharmingen (Franklin Lakes, NJ, USA).

### Mice

The randomized animal study included 110 male CB57/BL mice (between 20 and 30 g) housed and treated according to the Ministry of Social Justice and Empowerment (Government of China) guidelines (a temperature of 25 ± 1 °C, humidity of 60 ± 2%, and a 12 h light:dark cycle with free access to food and water). The animals were housed in standard mice cages with the usual mice bedding made of husk. Experiments were conducted between 08:00 and 14:00 h to minimize the diurnal variation. All procedures were approved by the local ethics committee of Sun Yat-sen University.

### PTX-induced seizure

PTX was dissolved in normal saline. Mice were injected intraperitoneally (i.p.) with PTX at a volume not exceeding 10 ml/kg. The mice were randomly divided into one control group and four treated groups (*n* = 30 in each group). The control group received normal saline (0.2 ml, i.p.), and the remaining four groups were injected with PTX. To determine the proper dose of PTX, the mice in the four treatment groups were administered a PTX dose of 0.25, 0.5, 1, and 2 mg/kg, respectively. Moreover, different doses of PTX were injected every 20 min until appearance of the first clonic seizure. Usually, it took 3–5 injections to a first seizure. The mice were separately placed in cages and the onset times of their seizures were observed for 60 min. Meanwhile, their seizure severity was assessed according to the method of Singh et al. [29] who rated the seizures on a 7-score scale (from 0 to 6), with the following scoring criteria: 0, no response; 1, ear and facial twitching; 2, 1 to 20 myoclonic body jerks in 10 min; 3, more than 20 body jerks in 10 min; 4, clonic forelimb convulsions; 5, generalized clonic convulsions with episodes of rearing and falling down; 6, generalized convulsions with tonic extension episodes. If the seizure score was higher than 3, the response to PTX was considered positive [30], and only those animals were used in the study. The onset time of seizure was recorded as 60 min when no seizure was observed in a mouse (seizure score was less than 3). The results for the treated groups were compared with those for the control group. Each mouse of the respective groups was placed in a separate cage. After being restrained for 6 h daily for 21 days [31], the mice were sacrificed by decapitation.

### Isolation and culture of microglial cells

Microglia of mouse brain tissues were obtained by using a modified version of the protocol described by Vinet [32]. Briefly, after the 21 days of observational study, mice were sacrificed by decapitation under ether anesthesia. The hippocampus of each brain was collected and put into ice-cold HBSS supplemented with 7.5 mM HEPES and 0.6% glucose (Sigma-Aldrich). Next, hippocampi were dissected, minced, and mechanically dissociated using a tissue homogenizer followed by a filtering step using a 70 μm cell strainer (BD Falcon, Bedford, MA, USA) to achieve a single-cell suspension [33]. To obtain a sufficient yield during cell sorting, the hippocampi of two animals were pooled together. Cells were pelleted and centrifuged at 220 rcf for 10 min (acc: 9, brake: 9, 4 °C). The supernatant was removed and the pellet resuspended in a solution of 22% Percoll (GE Healthcare, Chicago, IL, USA), 40 mM NaCl, and 77% myelin gradient buffer (5.6 mM NaH_2_PO_4_-H_2_O, 20 mM Na_2_HPO_4_-2H_2_0, 140 mM NaCl, 5.4 mM KCl, 11 mM glucose, pH 7.4). On top of this mix, a layer of phosphate-buffered saline (PBS) was added, and this gradient was centrifuged at 950 rcf for 20 min (acc: 4, brake: 0, 4 °C). Thereafter, the myelin layer and the remaining supernatant were carefully removed and the pellet resuspended in DMEM culture medium containing 10% FBS and 1% penicillin/streptomycin (Sigma-Aldrich) to yield the mixed cell suspension. The mixed cells were then seeded into poly-d-lysine pre-coated T75 tissue culture flasks (two brains/flask). After every 4 days of primary culture, the medium was changed. The cells were collected on day 12 by gently shaking the flasks for 30 min on an orbital shaker at 37 °C. Then, the supernatant were observed under a microscope. The cell suspension was collected and centrifuged at 500×*g* for 5 min. Cells were harvested and plated on poly-d-lysine-coated 12-well plates for 30 min. The plating medium was changed and non-adherent cells were removed. The primary microglia were used either for assays 1 week after plating or detached and re-seeded for in vitro assay.

### Cell sorting

Fc receptors were blocked with anti-mouse CD16/CD32 for 10 min on ice. Cells were then incubated for 30 min on ice with CD11b PE, CD45 FITC, and Ly-6C APC (Abcam) and subsequently washed with Medium A without Phenol Red and sorted using a BD FACSAria III cell sorter (Becton Dickinson, Franklin Lakes, NJ, USA). Microglia were defined as CD11b^pos^ CD45^int^ Ly-6C^neg^ [32] and determined by flow cytometry with a mean purity of 99%. Viability was tested using trypan blue exclusion dye. Finally, cells were suspended at 1 × 10^5^ cells/ml in DMEM supplemented with 10% FBS.

### RT-PCR

Microglia were collected from the seizure and control groups of mice at different time points after stimulation with or without LPS (1 μg/ml). Total RNA was extracted and the expression of IL-10 and IL-1β was detected by RT-PCR. Briefly, total RNA was purified with an RNAeasy mini kit (Tiangen, Beijing, China), and assessed for quality and quantity on a spectrophotometer (Beckman Coulter, Brea, CA, USA). Reverse transcription was carried out in 20 μl reaction volume with a High Capacity Reverse Transcription Kit (Applied Biosystems, Foster City, CA) following the manufacturer’s instructions. PCR was carried out on a DNA thermal cycler (Biometra, Germany) at 95 °C (5 min), 95 °C (45 s), 58 °C (45 s), and 72 °C (45 s), followed by 20–25 cycles of 72 °C for 5 min. GAPDH was used for normalization. The primers (TAKARA, Dalian, China) include GAPDH, 5′-AAATGGTGAAGGTCGGTGTGAAC-3′ (forward) and 5′-CAACAATCTCCACTTTGC CACTG-3′ (reverse); IL-10, 5′-GCCAGAGCCACATGCTCCTA-3′ (forward) and 5′-GATAAGG CTTGGCAACCCAAGTAA-3′ (reverse); IL-1β, 5′-TCCAGGATGAGGACATGAGCAC-3′ (forward) and 5′-GAACGTCACACACCAGCAGGTTA-3′ (reverse). PCR products were assessed by 1.5% agarose gel electrophoresis, and visualized with ethidium bromide under UV light.

### ELISA

The supernatants of IL-1β and IL-10 (BD Bioscience Pharmingen, Franklin Lakes, NJ, USA) were evaluated by ELISA kits according to the manufacturer’s protocol. Briefly, the ability of cells to secrete IL-1β in response to cytokines was determined using a sandwich ELISA. A flat-bottom 96-well microtiter plate (Greiner Bio-One, Kempten, Germany) was coated with 100 μl/well of anti-human IL-1β mAb (2 mg/ml in a mixture of sodium carbonate and sodium bicarbonate, pH 9.5) overnight at 4 °C. The plate was subsequently washed with phosphate-buffered saline (PBS; pH 7.0) and 0.05% Tween-20, and blocked with 10% fetal calf serum (FCS). IL-1β standards (rHu IL-1β) were made in a solution consisting of PBS (pH 7.0) and 10% FCS using serial dilutions. Standards or supernatants (100 μl/well) were plated in triplicate and incubated at ambient temperature for 2 h. After three washes, 100 μl/well of biotinylated anti-human IL-1β mAb (100 ng/ml in PBS, pH 7.0, and 10% FCS) was added, followed by 100 μl/well of streptavidin–peroxidase conjugate. The chromogen substrate was used at 100 μl/well; after 30 min, 10% H_2_SO_4_ was added to stop the reaction. Absorbance was read at 450 nm on an automated microplate reader (Bio-Tek Instruments, Richmond, CA, USA). IL-10 was also determined by ELISA using a specific ELISA kit.

### Western blotting

Cell lysis was carried out in a lysis buffer containing 50 mM Tris (pH 7.5), 1% (*v*/*v*) Triton X-100, 150 mM NaCl, 10% (*v*/*v*) glycerol, 1 mM EDTA, and a protease inhibitor cocktail. The resulting lysates were resolved by 10% SDS-PAGE, and then were transferred onto polyvinylidene difluoride (PVDF) membranes (Millipore, USA). These samples were incubated with mouse polyclonal antibodies (pAbs) and horseradish peroxidase-conjugated goat anti-mouse secondary antibodies (1:10000, Santa Cruz Biotechnology) overnight at 4 °C. The pAbs were raised against STAT-1, phospho-STAT-1, STAT-3, phospho-STAT-3, proIL-1β, procaspase-1, and caspase-1 (1:1000, Cell Signaling Technology). Then, we used anti-GAPDH mouse mAb (1:1000, Santa Cruz) for normalization. Finally, an ECL Western blotting analysis system (Cell Signaling Technology) was used for visualization.

### Intracellular cytokine and cell surface staining

Microglial cells were isolated from the brain tissue of a mouse model of seizures. After gene silencing in vitro, STAT-1 and STAT-3 siRNA cells were harvested and cultured with or without LPS(1 μg/ml) plus IL-10 for 12 h with brefeldin A (10 μg/ml, Sigma) supplemented for the final 5 h of treatment. Then they were fixed with 4% paraformaldehyde, and permeabilized in PBS supplemented with 0.1% saponin (Sigma), 0.1% BSA, and 0.05% NaN_**3**_ overnight at 4 °C. Next, the cells were stained by conjugated mAbs for the surface markers CD11b, CD45, Ly-6C, and the intracellular cytokine IL-1β for 20 min at 4 °C in the dark. Analysis was carried out by flow cytometry on a FACS Calibur (BD) in FlowJo version 6.0 (Tree Star Inc., Ashland, OR, USA).

### In vitro gene silencing

STAT-1 and STAT-3 were the target genes. The 12.5 nM, 25 nM, 50 nM, and 100 nM siRNA solutions were respectively added at 37 °C to 6-well plates containing 1 × 10^5^ microglial cells. The transfection medium contained DMEM supplemented by 10% FBS, and was incubated with LPS (1 μg/ml) for another 48 h. Amounts of phosphorylated STAT-1 and STAT-3 were assessed by Western blots after cell lysis. STAT-1 and STAT-3 bands were quantitated with ImageJ (NIH, Bethesda, MD, USA), and normalized by GAPDH.

### Statistical analysis

Seizure onset and scores were recorded as mean ± SEM. Two-tailed, unpaired Student’s *t* tests were performed in GraphPad Prism 5.0 (GraphPad, San Diego, CA, USA) to test the difference between groups. A two-way analysis of variance (ANOVA) with post-hoc Bonferroni-corrected *t* tests was used to compare inflammatory cytokine or inflammasome levels between all groups at various time points or under different stimulus conditions. A one-way ANOVA with post-hoc Tukey-corrected *t* test was used to compare differences in cytokine levels among experimental groups. For each statistical test, a parametric test was chosen. *P* < 0.05 and *P* < 0.01 were defined as significant and very significant, respectively.

## Results

### Picrotoxin-induced seizure model in mice

In a first preliminary experiment, the dose–response characteristics of PTX were investigated across a broad range of doses from 0.25 to 2 mg/kg. Our results showed that PTX might cause mice to develop a seizure response in a dose-dependent manner. In our study, seizure onset was observed in PTX-treated groups when the dose of PTX was 0.25 mg/kg. At this dose, we did not find seizures in the control group, which was administered saline. By comparing the treated groups with the control group, we found that the seizure scores and percentage of positive responders were significantly different when the dose of PTX was at 1 and 2 mg/kg. In particular, within a few minutes after repeated injection of doses of 1 mg/kg of PTX, ear and facial twitching, Straub tail, head nodding, and occasional clonic seizures occurred, but a status epilepticus with continuous seizure activity was only observed in two of six mice. When doses of 2 mg/kg of PTX were injected, up to five of the six mice developed status epilepticus and but the survival of animals exhibiting status epilepticus was higher compared with the group injected PTX doses of 1 mg/kg (Table 1). Therefore, the protocol with administration of PTX by injecting 2 mg/kg every 20 min until development of status epilepticus was used for all further experiments. In subsequent experiments, 50 mice were injected with PTX (2 mg/kg i.p.) until developing seizure. Out of these 50 mice, 41 (out of 42) survived after seizure (97.6%), no mice were non-responders, and eight died prior to entering seizure or during/after seizure (16%) (Table 2). The severity of seizure was evaluated using Singh’s classification, and only those mice that were classified higher than 3 were used in the study. In addition, as control, 30 mice were treated with saline.

**Table 1:** Effect of PTX on seizure in mice

Dose of PTX (mg/kg, i.p.)	Number of mice tested	Mice with status epilepticus	Mice surviving status epilepticus	Seizure score ≥ 3	Total mortality
Saline control	6	0	0	0	0
0.25	6	0	0	0	0
0.5	6	0	0	0	0
1	6	2 (33.3%)*	1 (50%)	1 (16.7%)	1 (16.7%)
2	6	5 (83.3%)**	4 (80%)	4 (66.7%)	1 (16.7%)

Effects of PTX on status epilepticus mice. (*n* = 6 in each group; *P < 0.05 and **P < 0.01 compare the PTX-treated with the saline control group for the corresponding parameters)

**Table 2:** Results from the study of this investigation on PTX (2 mg/kg) in mice

Dose of PTX (mg/kg, i.p.)	Seizure score (mean ± SEM)	Number of + ve responder/total (% of + ve responders)	Time to onset of status epilepticus (min) (mean ± SEM)	Mice surviving status epilepticus	Total mortality	Mice developing spontaneous seizures aster epilepticus
Saline control	0	0/30 (0)	60 ± 0	30	0	0
2	4.5 ± 1.0	48/50 (96%)**	3.3 ± 1.0	42 (87.5%)	8 (16%)	41 (97.6)

The effect of PTX on the frequency of epilepsy with spontaneous recurrent seizures developing in mice was evaluated. (*n* = 30 in the saline control group and *n* = 50 in the PTX-treated group; **P < 0.01 compares the PTX-treated and the saline control groups for the corresponding parameters)

### Microglial cells from epileptic mice spontaneously expressed IL-10 and IL-1β

**Fig. 1:**
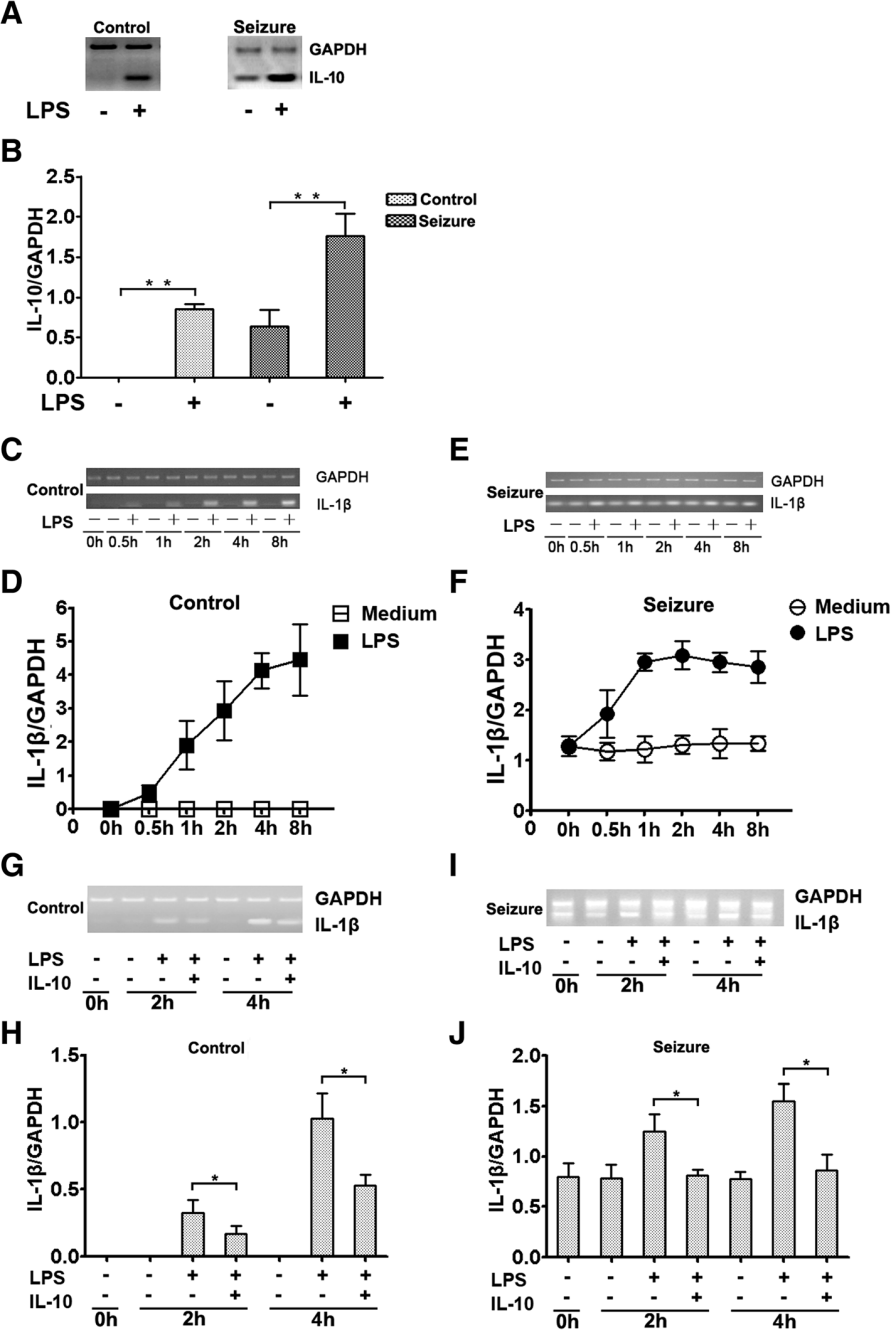
The expression of IL-10 and IL-1β in microglia. **a** Microglia from mice in the control group (*n* = 6) and epileptic-seizure mice (*n* = 6) were incubated with or without LPS (1 μg/ml), and IL-10 mRNA was evaluated by RT-PCR. **b** Graphs show the ratios of IL-10/GAPDH. Data show mean ± SEM of four separate experiments. **c**, **e** The expression of IL-1β in LPS-stimulated microglia of mice from the control group (*n* = 6) and epileptic-seizure mice (*n* = 6). The level of IL-1β was assessed by RT-PCR. **d**, **f** Statistical results for the IL-1β/GAPDH ratios. Data represent mean ± SEM of four independent experiments. GAPDH was used for normalization. **P < 0.01. **g**, **i** Microglial cells from the control group of mice (**g**) (*n* = 6) and epileptic-seizure mice (**i**) (*n* = 6) were treated with or without LPS (1 μg/ml) in the presence or absence of exogenous IL-10, and the expression of IL-1β was measured by RT-PCR. **h**, **j** Quantitative data are mean ± SEM from three independent experiments. *P < 0.05

To investigate the effect of IL-10 and IL-1β in the process of seizure, we performed a series of experiments directly detecting the expression levels of IL-10 and IL-1β in microglia. Microglia were collected at different time points after stimulation with or without LPS (1 μg/ml) from the seizure and control groups of mice. Total RNA was extracted and the expression of IL-10 and IL-1β was detected by RT-PCR. Interestingly, we found that IL-10 (Fig. 1a) and IL-1β (Fig. 1c, e) were expressed in freshly isolated microglia from epileptic seizure mice but not in the control group. Moreover, the expression of IL-1β significantly increased in a time-dependent manner after stimulation with LPS in both the control group and the epileptic-seizure mice (Fig. 1c, e). However, as the incubation time increased, the level of IL-1β started to become stable (Fig. 1e). Quantitative data on IL-10 and IL-1β are shown in Fig. 1b, d, f, respectively; they were calculated by dividing the expression levels of IL-10 and IL-1β by the amounts of GAPDH in microglia after stimulation with LPS. The expression levels of IL-10 and IL-1β were not found to increase at higher doses of LPS (data not shown).

To assess the anti-inflammatory effects of IL-10 on the expression of IL-1β, microglial cells from mice of the control group and with epileptic seizures were incubated with IL-10 at different time points, and IL-1β mRNA levels were evaluated by RT-PCR. The results indicated that the expression of IL-1β was reduced in both the control group (Fig. 1g) and in epileptic-seizure mice (Fig. 1i) after stimulation with LPS (1 μg/ml) in the presence of IL-10. The graphs show the ratios of IL-1β expression levels (Fig. 1h, j) to GAPDH.

### IL-10 regulated the production of IL-1β

**Fig. 2:**
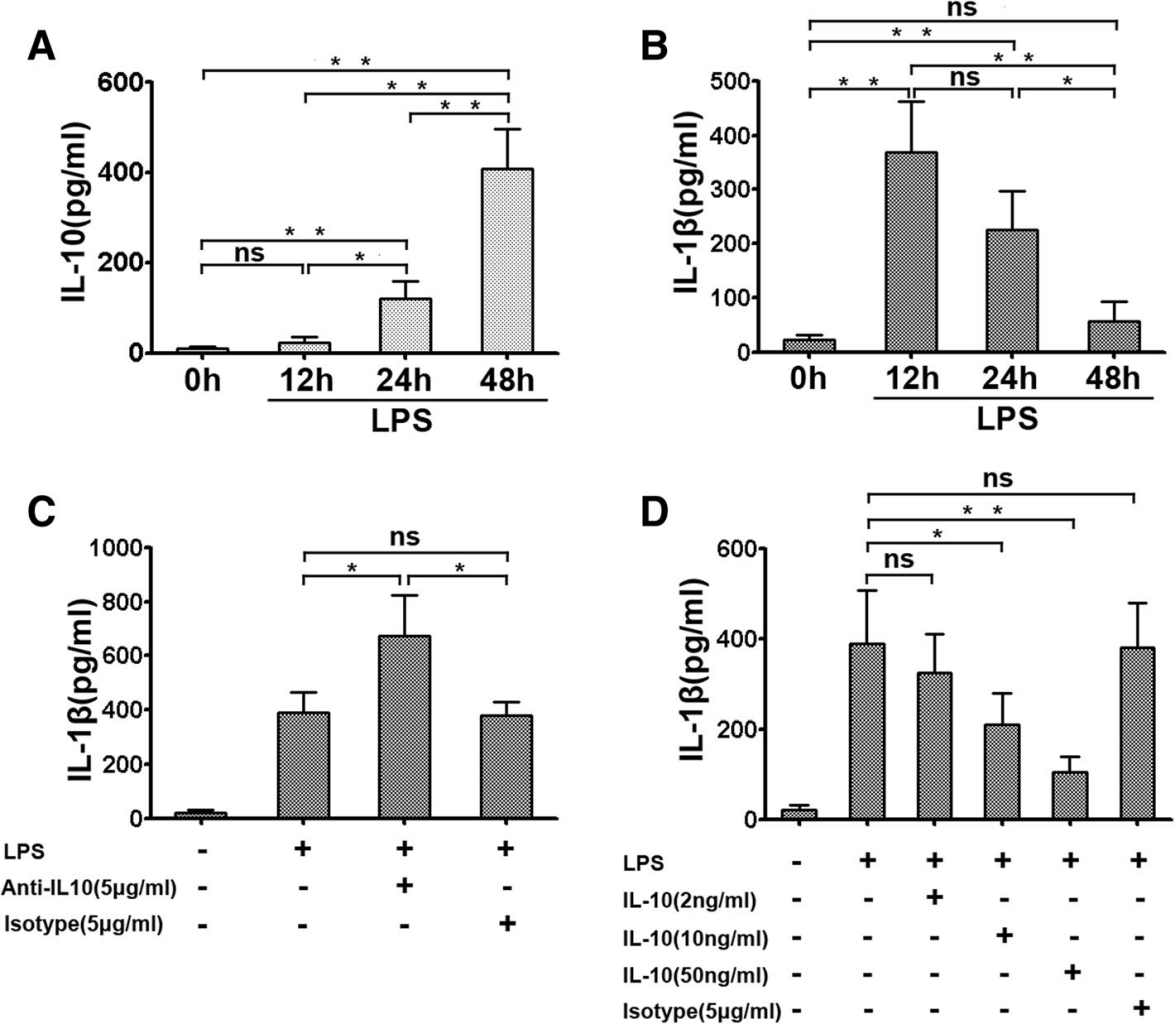
IL-10 inhibits the production of IL-1β by microglial cells. **a**, **b** The secretion of IL-10 and IL-1β were assessed by ELISA in the supernatants of microglial cells from epileptic-seizure mice after LPS stimulation at different time points (*n* = 6). **c** Endogenous IL-10 suppressed IL-1β production by microglia during LPS stimulation (*n* = 6). Microglial cells were activated with LPS in the presence or absence of IL-10-neutralizing antibodies at different doses or of isotype-matched control antibodies for 12 h. The level of IL-1β was evaluated by ELISA. **d** Microglial cells were incubated with medium, LPS, increasing concentrations of IL-10, or isotype-matched control antibodies for 12 h. The level of IL-1β in supernatants was detected by ELISA (*n* = 6). All statistical results are shown as mean ± SEM from four independent experiments. *P < 0.05; **P < 0.01; *ns* not significant

To investigate the contribution of IL-10-mediated IL-1β in microglia from seizure mice, we examined whether IL-10 affects the production of IL-1β at the protein level. After LPS (1 μg/ml) stimulation for 12 h, 24 h, and 48 h, the levels of IL-10 and IL-1β in cell-free culture supernatants were determined by ELISA. We found that the production of IL-10 was significantly increased by stimulation with LPS in a time-dependent manner. When incubated for 48 h, the level of IL-10 was the highest (Fig. 2a). In contrast, the secretions of IL-1β were significantly reduced as time progressed (Fig. 2b). To evaluate the effects of IL-10 on IL-1β production, microglia from seizure mice were cultured in the presence of IL-10-neutralizing antibodies or isotype-matched control antibodies under LPS (1 μg/ml) stimulation for 12 h. Our results showed that IL-10 neutralization resulted in markedly increased IL-1β levels, while the isotype control group showed no change (Fig. 2c) (*P < 0.05).

To further evaluate the impact of exogenous IL-10 on IL-1β, microglia from epileptic seizure mice were treated with IL-10 at different concentrations in combination with LPS (1 μg/ml) for 12 h. The secretion of IL-1β in the supernatants was measured by ELISA. We found that the level of IL-1β was significantly reduced than stimulated with LPS alone. The optimal IL-10 concentration for IL-1β inhibition was 50 ng/ml. In comparison, there was no effect on an isotype-matched control antibody (Fig. 2d) (*P < 0.05, **P < 0.01).

### IL-10 inhibits IL-1β production in a STAT-3-dependent manner

**Fig. 3:**
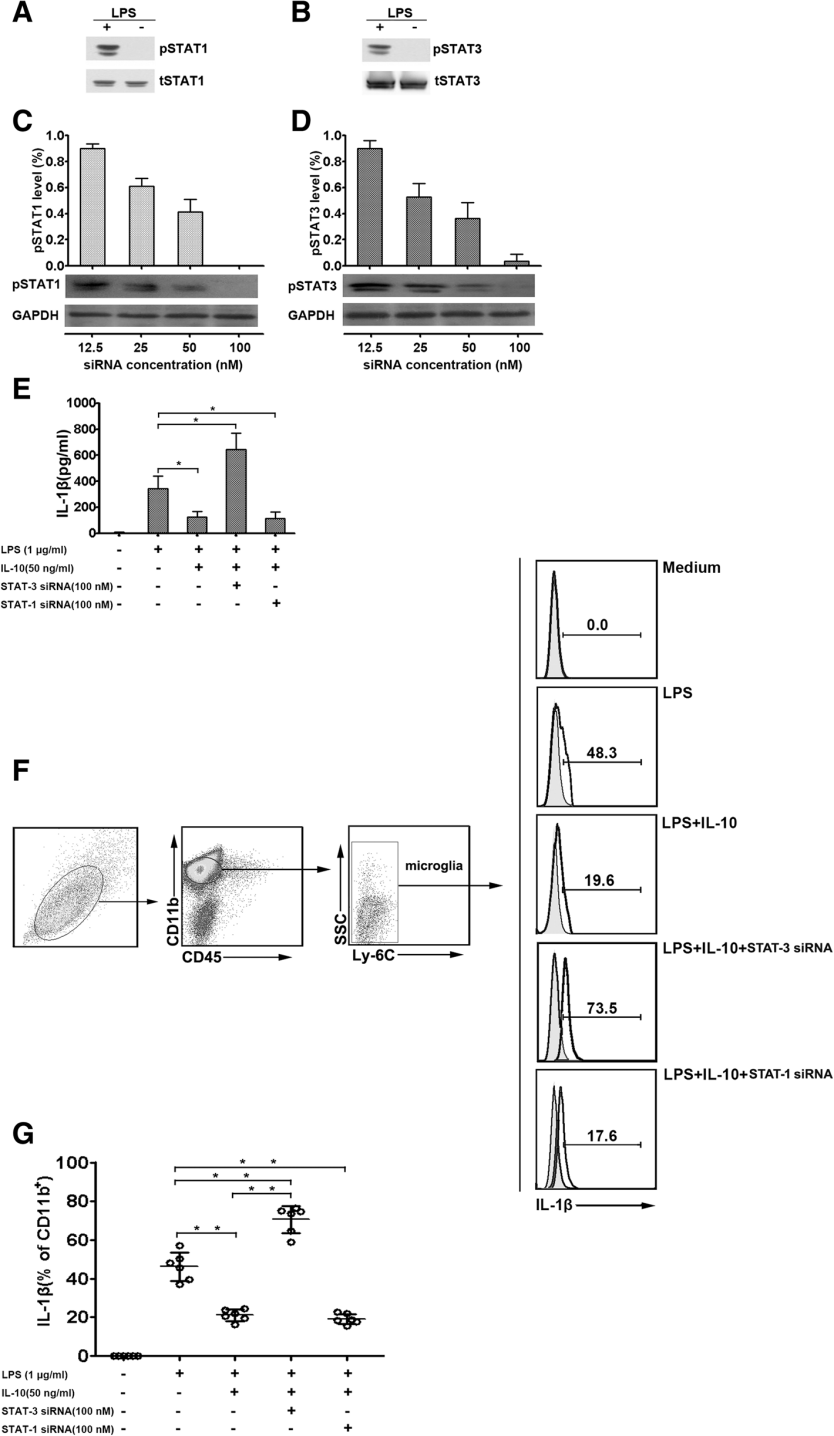
The production of IL-1β via IL-10 and STAT-3. **a**, **b** STAT-1 and STAT-3 phosphorylation in microglia. Microglial cells from epileptic-seizure mice were incubated for 8 h with or without LPS, and phosphorylated STAT-1 (**a**) and STAT-3 (**b**) in cell lysates were measured by western blot (*n* = 6). The results are representative of three independent experiments. **c**, **d** Microglial cells from epileptic-seizure mice were incubated for 8 h with STAT-1 and STAT-3 siRNAs. Then, LPS was added to activate the microglial cells. Western blot analysis showing a dose-dependent reduction of pSTAT-1 (**c**) and pSTAT-3 (**d**) signals in cell lysates (*n* = 6). The graphs show the ratios of pSTAT-1 or pSTAT-3 over GAPDH. Data indicate mean ± SEM of three individual experiments. GAPDH served as an internal control. **e** The production of IL-1β was assessed by ELISA in supernatants of microglia from epileptic-seizure mice after STAT-1 or STAT-3 knockdown and stimulation with LPS for 12 h (*n* = 6). Data represent mean ± SEM from three independent experiments.*P < 0.05. **f** IL-1β-positive cells were evaluated by flow cytometry in microglia from epileptic-seizure mice after LPS stimulation for 8 h in the presence or absence of IL-10 (*n* = 6). Numbers represent percentages of cells in various tests. **g** Data represent mean ± SEM from four independent experiments. *P < 0.05; **P < 0.01

STATs are crucial for IL-1β downregulation by IL-10 [34–36]. To assess the direct effects of IL-10 on STAT-1 or STAT-3 phosphorylation, pSTAT-1 and pSTAT-3 levels in microglial cells from epileptic seizure mice were assessed in the presence of LPS. Our results showed that both phosphorylated STAT-1 and STAT-3 were significantly increased in microglia after stimulation with LPS (1 μg/ml) (Fig. 3a, b). Next, we evaluated the effects of specific siRNAs on STAT-1 and STAT-3 knockdown in microglial cells from epileptic-seizure mice. We found that both STAT-1 and STAT-3 siRNAs specifically downregulated STAT-1 and STAT-3 in a dose-dependent manner after stimulation with LPS (1 μg/ml) (Fig. 3c, d). This result suggested that STAT-1 and STAT-3 activation was completely inhibited in vitro. In addition, both STAT-1 and STAT-3 gene silencing control experiments are shown in Additional file 1: Figure S1.

To explore the mechanisms behind the crosstalk between IL-10 and IL-1β, the effects of IL-10 on LPS-induced IL-1β production under different culture conditions were evaluated. Our results showed that when STAT-3 expression was reduced by its specific siRNA in microglia, IL-10 could no longer inhibit the production of IL-1β. In contrast, IL-1β secretion showed no significant difference between cells incubated with IL-10 after LPS (1 μg/ml) induction and those treated with STAT-1 siRNA (Fig. 3e). The roles of STAT-1 and STAT-3 on IL-1β cytokine production at the single-cell level were assessed by flow cytometry, which detected the level of IL-1β in microglial cells from epileptic mice. Consistent with the data of ELISA, microglial cells from epileptic-seizure mice incubated with IL-10 were significantly less frequently IL-1β-positive than incubated cells in the LPS alone group. In addition, IL-10 did not universally downregulate IL-1β production after STAT-3 siRNA treatment. Moreover, compared with LPS and IL-10 co-stimulated microglial cells, no significant difference was observed in the number of IL-1β-positive cells after STAT-1 siRNA knockdown (Fig. 3f). The statistical results show the level of IL-1β-positive cells (Fig. 3g). These findings suggest that IL-10 reduces IL-1β production in a STAT-3-dependent manner.

### IL-10 suppresses both caspase-1 maturation and proIL-1β availability

**Fig. 4:**
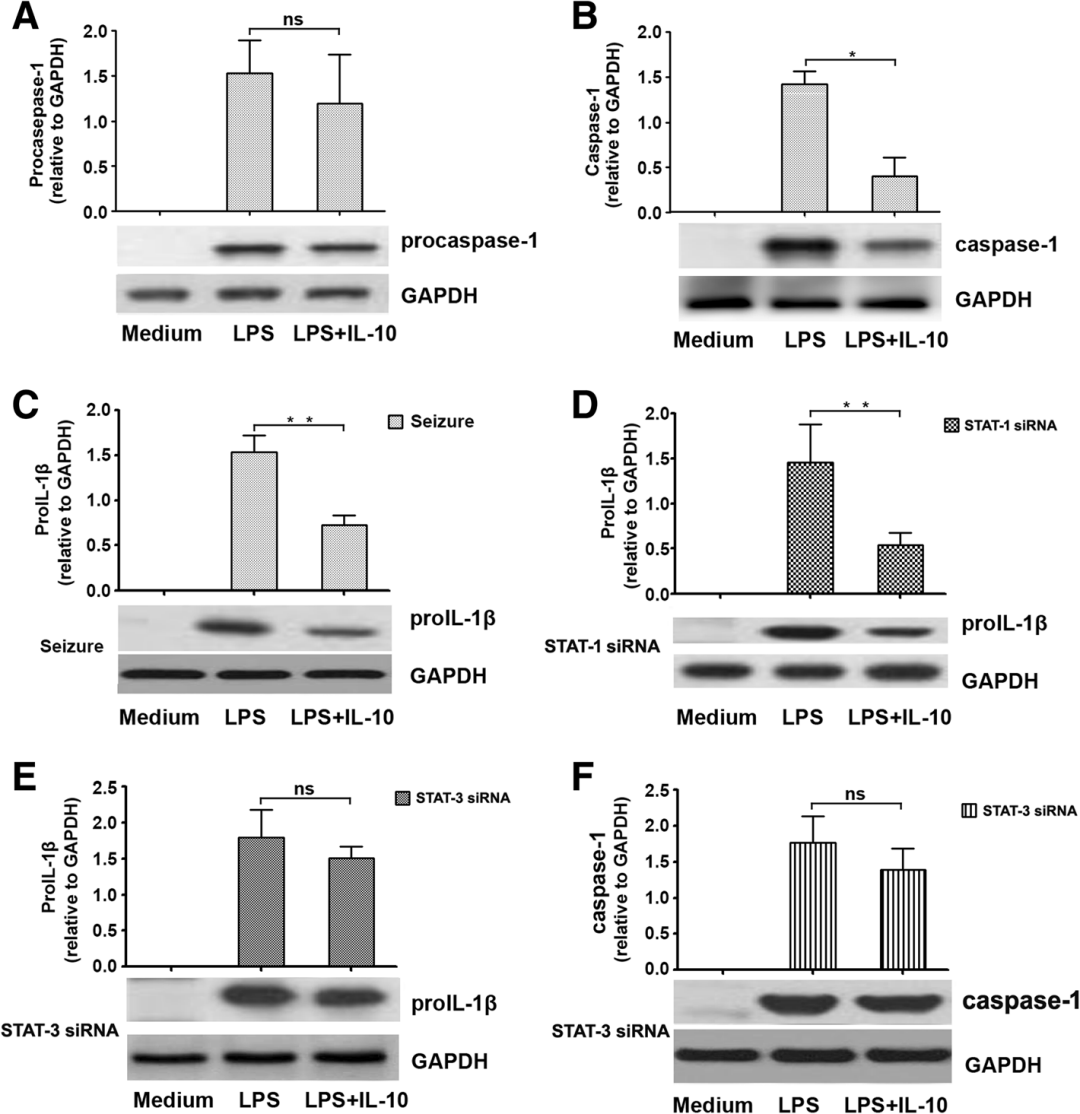
IL-10 reduces inflammasome activity and proIL-1β levels. **a**, **b** Microglia were incubated for 8 h with or without LPS. Amounts of procaspase-1 and caspase-1 were determined by Western blotting (*n* = 6). Expression levels of procaspase-1 and caspase-1 were normalized to amounts of GAPDH, and data represent mean ± SEM of four individual experiments; *ns* not significant. *P < 0.05. **c**–**e** IL-10 regulates IL-1β precursor levels. Microglial cells from epileptic seizure mice, STAT-1 and STAT-3 siRNA knockdown, respectively, were treated with LPS in presence or absence of exogenous IL-10. ProIL-1β levels were assessed in cell lysates (*n* = 6). **P < 0.01, *ns* not significant. **f** Microglial cells from STAT-3 siRNA knockdown mice were stimulated with LPS in the presence or absence of exogenous IL-10. Caspase-1 activation was assessed by Western blot (*n* = 6). A representative Western blot is shown from three independent experiments. *ns* not significant

To evaluate the anti-inflammatory effects of IL-10 on IL-1β production, microglia were incubated for 8 h with LPS (1 μg/ml) in the presence/absence of IL-10. Procaspase-1 and caspase-1 levels in cell lysates were measured by Western blotting. We found that procaspase-1 inflammasome activation was not inhibited after stimulation with IL-10 (Fig. 4a), but did not observe the same result in caspase-1 (Fig. 4b). To further evaluate the impact of IL-10 on IL-1β production, the availability of proIL-1β was assessed. The results showed that IL-10 played a crucial role in the negative regulation of proIL-1β in microglial cells both from epileptic mice (Fig. 4c) and STAT-1-silenced mice (Fig. 4d). Meanwhile, proIL-1β levels were not reduced after STAT-3 silencing in mice (Fig. 4e). We next assessed whether IL-10 could suppress caspase-1 production in cells after STAT-3 silencing in the presence of IL-10, and found that secreted caspase-1 levels were not significantly different compared with those of cells stimulated with LPS (1 μg/ml) alone (Fig. 4f). These findings demonstrate that IL-10 regulates IL-1β in two ways, by inhibiting the inflammasome function and by reducing intracellular proIL-1β levels.

### IL-10 reduces NLRP3 inflammasome activation in a STAT-3-dependent manner

**Fig. 5:**
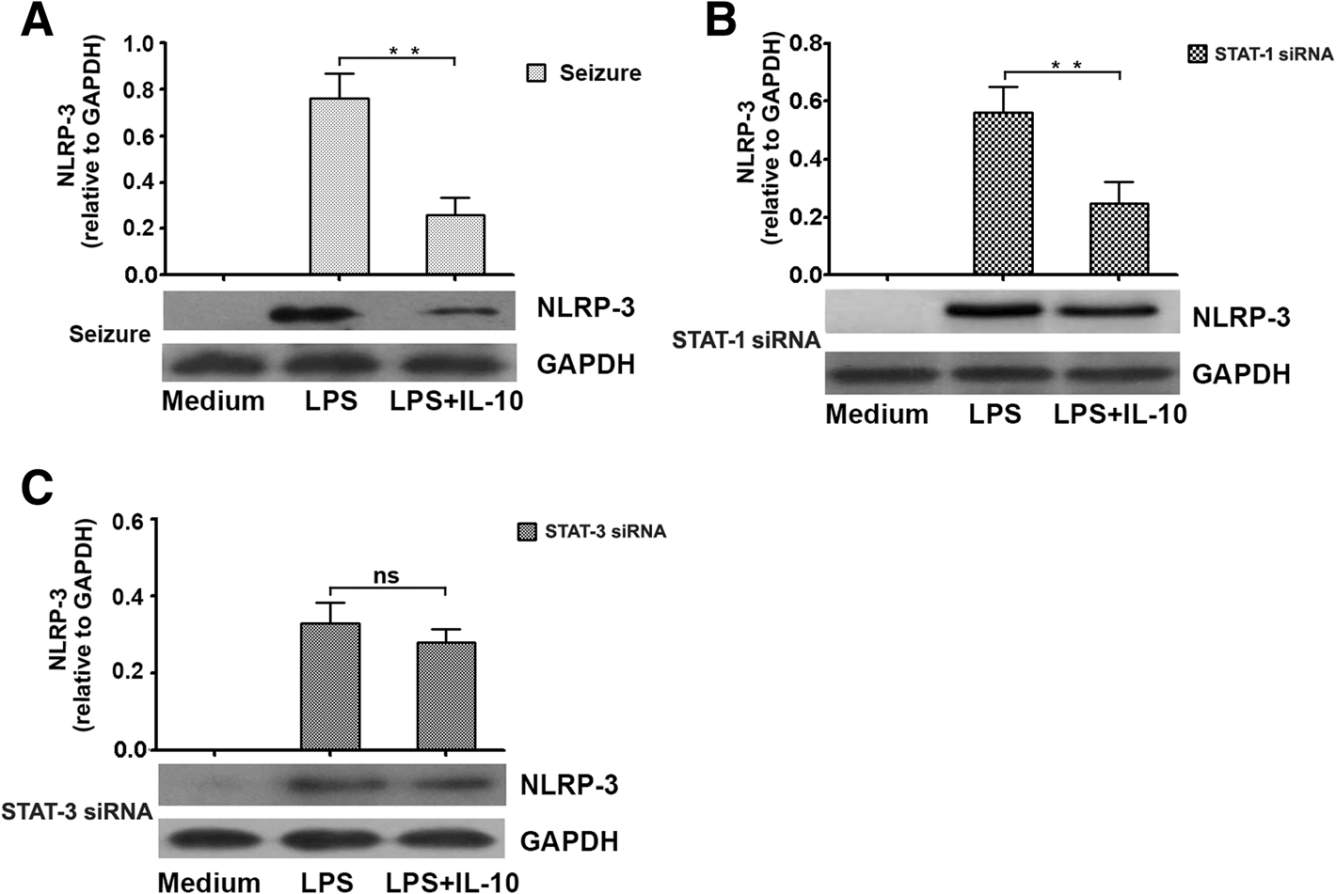
IL-10 modulates the STAT-3 pathway to suppress NLRP-3 expression by microglia. **a**–**c** Microglia from epileptic-seizure mice, STAT-1 siRNA mice, or STAT-3 siRNA mice were incubated for 8 h with LPS. NLRP-3 levels in cell lysates were measured by Western blots (*n* = 6). Data represent mean ± SEM of four individual experiments. **P < 0.01; *ns* not significant

Previous studies indicated that different reaction conditions could induce IL-1β production by activating the NLRP3 inflammasome [37, 38]. However, the mechanisms by which IL-1β regulates the host’s response to seizures are poorly understood in both mice and humans. So, we tested the inhibitory effects of IL-10 in NLRP-3 inflammasome. We first compared NLRP-3 expression levels in microglial cells of the epileptic-seizure mice with those in STAT-1 siRNA and STAT-3 siRNA groups treated with or without IL-10. Interestingly, significantly decreased NLRP-3 inflammasome activation in the epileptic seizure mice (Fig. 5a) and in the STAT-1 siRNA group were observed after stimulation with IL-10 (Fig. 5b). However, IL-10 was unable to inhibit NLRP-3 inflammasome activation in cells from the STAT-3 siRNA group (Fig. 5c). These findings suggest that IL-10 inhibits NLRP-3 inflammasome activation in a STAT-3-dependent manner.

## Discussion

Up to 1% of humans are suffering from epilepsy, with approximately 35% of cases displaying identifiable CNS injury [39]. To date, no available therapeutic options prevent or reverse the progression of epileptic disease. Recently, inflammatory processes in cerebral tissues were reported in patients with epilepsy of distinct etiologies as well as in animal models of seizure [40]. In general, inflammation promotes the development of seizures, and vice versa. Meanwhile, inflammation seems to promote epileptogenesis even in the developing brain. An acute inflammatory response to an insult is largely a protective cellular response. Lipopolysaccharide (LPS) is one of the many factors that cause inflammation [41]. LPS is recognized by microglia and binds to cell surface receptors such as Toll-like receptors-4 (TLR-4). Then they trigger receptor dimerization at the plasma membrane, which, in turn, activates signal-transduction cascades to induce inflammation [42]. Activation of TLR-4 can initiate different inflammatory responses, including inducing the expression of proinflammatory cytokines, and triggers the formation of inflammasomes [26]. Nod-like receptor protein-3 (NLRP-3) inflammasome [43, 44] is one of the inflammasomes and contains the precursor pro-caspase-1, which is cleaved triggered by inflammatory stimuli to release its active form, caspase-1 [45]. Caspase-1 is a cysteine protease that converts Pro-IL-1β to the active cytokine IL-1β, which is then released extracellularly to propagate inflammatory signals.

Studies have suggested that inflammatory reactions result in worsened consequences of epilepsy in animal models [46]. In particular, high levels of proinflammatory cytokine (IL-1β and TNF-α) are found in epileptogenic tissues from mice with epilepsy of various etiologies [47, 48]. Moreover, after tonic–clonic seizures, in vivo studies indicated an elevated production and secretion of proinflammatory cytokines like IL-1β, IL-6, and TNF-α in cerebral spinal fluid (CSF) and blood serum in patients with epilepsy [2, 49].

It is known that IL-1β and TNF-α can be released by microglial cells and influence neuronal excitability; therefore, these cytokines are under intense investigation for their possible contributions to epileptogenesis [9, 50–53]. Meanwhile, microglial cells also express the anti-inflammatory cytokines IL-4 and IL-10 [54]. Moreover, the effects of IL-10 in immune and inflammatory responses were reported before, and they were described as reduced levels in certain inflammatory diseases [55–57]. However, the mechanisms by which IL-10 regulates IL-1β production and inflammasome activation in microglia with epilepsy have not been established. A better understanding of the specific immune responses and functional mechanisms regulating this anti-inflammatory cytokine may help develop new therapies for epilepsy.

To determine the function of IL-10 in secreting IL-1β, we first analyzed the expressions of IL-10 and IL-1β in microglial cells from epileptic-seizure mice and compared them with mice from the control group by RT-PCR. Our results indicate that microglial cells from epileptic mice express IL-10 and IL-1β in vivo while mice from the control group did not show expression of these two genes. Furthermore, this study demonstrates that IL-10 may exert its anti-inflammatory effect, at least in part, by regulating IL-1β production. Levels of IL-1β, whose maturation relies on caspase-1, were shown to be decreased in microglia treated with IL-10. Meanwhile, the activation of IL-1β secretion is countered by either reducing pro-IL-1β protein or inhibiting NLRP3 inflammasome-dependent caspase-1. The predominant mechanism leading to IL-10-related inhibition of pro-IL-1β depends on the STAT-3-mediated synergistic effect of LPS for upregulating IL-10. In agreement with previous findings, LPS specifically activates IL-10, in line with the above data showing that IL-10 significantly decreases the level of pro-IL-1β. In vitro, LPS triggered a strong induction of IL-10 that efficiently prevented pro-IL-1β expression. Hence, the balance between IL-10 induction and amounts of pro-IL-1β could determine the final amounts of mature IL-1β. In addition, STAT-3 is important in NLRP-3 inflammasome inhibition by IL-10. Our findings demonstrate that STAT-3 phosphorylation was sufficient for suppressing NLRP3 inflammasome activity, indicating that this inhibitory pathway, which is probably intrinsic to cells, needs to be disclosed in further studies.

Although many previous studies have found that IL-1β contributes to epileptic seizures, with IL-10 showing anticonvulsant effects [58, 59], few reports have clarified which periods of a seizure are influenced. In this study, we described complex pro- and anti-inflammatory responses during epileptogenesis, and first demonstrated that freshly isolated microglia from epileptic-seizure mice but not those of control group of mice spontaneously expressed IL-10 and IL-1β. Meanwhile, exogenous IL-10 significantly reduced the level of IL-1β in microglia after LPS induction. However, additional studies are required to unveil the exact role of inflammation in epilepsy. As shown above, neutralization of IL-10 by specific antibodies resulted in significantly increased secretion of IL-1β. Importantly, we indicated that IL-10 inhibits IL-1β production via IL-10 signaling and the STAT-3 transcription factor, and by suppressing caspase-1-dependent IL-1β maturation; in addition, autocrine IL-10 could reduce the abundance of pro-IL-1β. These findings suggest that IL-10 uses different pathways to regulate the level of IL-1β, and reveal that IL-10 induction might be associated with disease progression in patients with seizure.

## Conclusions

Overall, IL-10 exerts its function in the progression of seizure by inhibiting IL-1β production via two mechanisms. To begin, IL-10 could activate STAT-3, which suppresses the caspase-1-dependent maturation of IL-1β; further, autocrine IL-10 could reduce the abundance of pro-IL-1β directly. These findings suggest that IL-10 might alleviate symptoms, influencing disease progression in epilepsy patients.

## Additional file

Additional file 1: **Figure S1.** Gene silencing control experiments of STAT-1 and STAT-3 phosphorylation in microglia. (A) Microglial cells from epileptic-seizure mice were incubated for 8 h without STAT-1 and STAT-3 siRNAs. Then, LPS was added to activate the microglial cells. Western blot analysis showing pSTAT-1 and pSTAT-3 signals in cell lysates (*n* = 6). (B) Graphs showing the ratios of pSTAT-1 or pSTAT-3 over GAPDH. Data represent mean ± SEM of three individual experiments. GAPDH served as an internal control. (TIF 962 kb)

## Abbreviations

APC: Allophycocyanin
CNS: Central nervous system
DMEM: Dulbecco’s modified Eagle’s medium
ELISA: Enzyme-linked immunosorbent assay
FACS: Fluorescence-activated cell sorter
FBS: Fetal bovine serum
FCS: Fetal calf serum
FITC: Fluorescein isothiocyanate
GAPDH: Glyceraldehyde-3-phosphate dehydrogenase
HBSS: Hank’s balanced salts solution
HEPES: 4-(2-Hydroxyethyl)-1-piperazineethanesulfonic acid
IL: Interleukin
LPS: Lipopolysaccharide
NLRP3: NOD-like receptor family pyrin domain containing 3
PBS: Phosphate-buffered saline
PE: Phycoerythrin
PTX: Picrotoxin
PVDF: Polyvinylidene difluoride
RT-PCR: Reverse transcription-polymerase chain reaction
STAT: Signal transducer and activator of transcription
TLR: Toll-like receptor
TNF: Tumor necrosis factor
