# Supplementary figures and images for: Interleukin-10 inhibits interleukin-1β production and inflammasome activation of microglia in epileptic seizures
Source: J Neuroinflammation. 2019 Mar 28;16:66. doi: 10.1186/s12974-019-1452-1 (PMC6437919; doi:10.1186/s12974-019-1452-1)

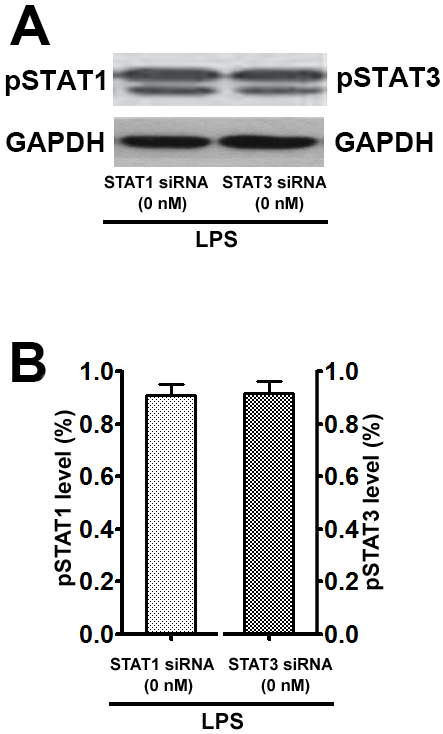

Supplement: Supplementary file 1 — Figure S1. Gene silencing control experiments of STAT-1 and STAT-3 phosphorylation in microglia. (A) Microglial cells from epileptic-seizure mice were incubated for 8 h without STAT-1 and STAT-3 siRNAs. Then, LPS was added to activate the microglial cells. Western blot analysis showing pSTAT-1 and pSTAT-3 signals in cell lysates (n = 6). (B) Graphs showing the ratios of pSTAT-1 or pSTAT-3 over GAPDH. Data represent mean ± SEM of three individual experiments. GAPDH served as an internal control. (TIF 962 kb) [file 12974_2019_1452_MOESM1_ESM.tif]
